# Tissue-specific features of the X chromosome and nucleolus spatial dynamics in a malaria mosquito, *Anopheles atroparvus*

**DOI:** 10.1371/journal.pone.0171290

**Published:** 2017-02-03

**Authors:** Semen M. Bondarenko, Gleb N. Artemov, Igor V. Sharakhov, Vladimir N. Stegniy

**Affiliations:** 1 Laboratory for Ecology, Genetics and Environmental Protection, Tomsk State University, Tomsk, Russia; 2 Department of Entomology, Fralin Life Science Institute, Virginia Polytechnic Institute and State University, Blacksburg, VA, United States of America; New Mexico State University, UNITED STATES

## Abstract

Spatial organization of chromosome territories is important for maintenance of genomic stability and regulation of gene expression. Recent studies have shown tissue-specific features of chromosome attachments to the nuclear envelope in various organisms including malaria mosquitoes. However, other spatial characteristics of nucleus organization, like volume and shape of chromosome territories, have not been studied in *Anopheles*. We conducted a thorough analysis of tissue-specific features of the X chromosome and nucleolus volume and shape in follicular epithelium and nurse cells of the *Anopheles atroparvus* ovaries using a modern open-source software. DNA of the polytene X chromosome from ovarian nurse cells was obtained by microdissection and was used as a template for amplification with degenerate oligo primers. A fluorescently labeled X chromosome painting probe was hybridized with formaldehyde-fixed ovaries of mosquitoes using a 3D-FISH method. The nucleolus was stained by immunostaining with an anti-fibrillarin antibody. The analysis was conducted with TANGO—a software for a chromosome spatial organization analysis. We show that the volume and position of the X chromosome have tissue-specific characteristics. Unlike nurse cell nuclei, the growth of follicular epithelium nuclei is not accompanied with the proportional growth of the X chromosome. However, the shape of the X chromosome does not differ between the tissues. The dynamics of the X chromosome attachment regions location is tissue-specific and it is correlated with the process of nucleus growth in follicular epithelium and nurse cells.

## Introduction

Interphase chromosomes maintain integrity and occupy specific volume known as chromosome territories (CTs) inside the nucleus [1–2]. Non-random organization of CTs is important for the functioning of the genetic apparatus of the cell [3]. A significant aspect of the nuclear architecture is interaction between chromatin and other nuclear compartments. For example, lamina plays a fundamental role in the process of CT formation at the nuclear periphery [4]. The nucleolus is a ribosomal RNA synthesis center, which is formed by nucleolus organizer regions (NORs) localized on acrocentric chromosomes of humans [5] or on the X chromosome of fruit flies [6] and mosquitoes [7]. CTs that contain NORs usually localize near the nucleolus or associate with it [8]. Other chromosomal regions besides NORs known as nucleolus associated domains (NADs) may contact with the nucleolus as well [1].

CTs have tissue-specific features that have been associated with functional aspects of spatial organization of the interphase nucleus [9–10]. Some lamin-associated domains (LADs) differ between cell types while others are common to different cell types in mammals [11]. Attachments of polytene chromosomes to the nuclear envelope (NE) occur via heterochromatic regions and have tissue-specific differences in *Drosophila melanogaster* [12–13] and in *Anopheles* mosquitoes from the *Maculipennis* group [14–16]. Studying spatial organization of CTs and nucleoli would be important for understanding the spatial organization of transcription inside the cell nucleus in malaria mosquitoes. Knowledge about nuclear architecture in vectors of infectious diseases will provide a rich basis for fundamental and applied research aimed at deciphering the mechanisms controlling development and reproduction [17]. We have identified significant differences in interpositions of X and 3R chromosomes in several types of somatic and germ-line cells in *Anopheles messeae* [14,16]. On average, the X chromosome and 3R chromosome are located closer to each other in follicular epithelium cells (FE) in comparison with their location in ovarian nurse cells (NC). The imaginal disc cells nuclei have an intermediate arrangement of chromosome interposition, similar to that of other somatic cells and nurse cells [16].

In this work, we studied several aspects of nuclear architecture using ovarian follicles of *An*. *atroparvus* malaria mosquitoes that contain cells of both germ-line NC and somatic FE systems. This species was chosen for this study because some aspects of the spatial organization of chromosomes in the *Macullipennis* complex, to which *An*. *atroparvus* belongs, have been studied previously [14, 15]. Importanly, *An*. *atroparvus* is a vector of malaria in Europe and the only species in the *Macullipennis* group with sequenced and physicaly mapped genome [17].

Our study focused on spatial organization of the X chromosome because it is the shortest polytene chromosome in the set, and it is not as curved as the autosomes. These characteristics made the X chromosome more accessible for our study of the CT by simple geometrical quantitative measurements. Furthermore, the X chromosome of *An*. *atroparvus* contains NOR(s) allowing estimation of dynamics of size and location of the nucleolus in connection with spatial reorganization of the X chromosome in different tissues. In addition, we tested the application of novel methods of analysis in studying spatial organization of chromosomes in the ovaries of malaria mosquitoes.

## Materials and methods

### Mosquito colony and chromosome preparation

The *An*. *atroparvus* Tomsk laboratory colony was used for the described experiments. Mosquitoes were raised in the insectary at 24°C, with a 12-hour cycle of light and darkness. Ovaries of *An*. *atroparvus* half-gravid females were dissected and fixed in Carnoy’s fixative solution (75% ethanol, 25% acetic acid). For making preparations of polytene chromosomes from ovarian nurse cells, a single ovary from one pair was taken. Ovaries were incubated in a drop of 50% propionic acid for 5 minutes, macerated, and squashed. The quality of the chromosomal preparation was checked by AxioImager A1 microscope (Carl Zeiss, OPTEC Company, Siberian Office, Novosibirsk, Russia). High-quality preparations were frozen in liquid nitrogen. Preparations were dehydrated in a series of ethanol (50%, 70%, 90%, and 100%) and air dried. These chromosomal preparations were used for X chromosome microdissection and 2D-FISH.

### Microdissection of the X chromosome

We conducted microdissection of the *An*. *atroparvus* X chromosome using the technique described in previous work [18]. Polytene chromosomes were collected from the surface of air-dried preparations with the help of a glass capillary (Narishige, Tokyo, Japan) and inverted microscope Axiovert 200 (Carl Zeiss, OPTEC Company, Siberian Office, Novosibirsk, Russia). The collected material was incubated in proteinase K followed by reprecipitation in 96% ethanol and washing with 70% ethanol. Precipitated DNA was amplified by low-temperature cycles of PCR in the presence of sequenase (Sequenase Version 2.0, Affymetrix USB, Dia-m, Novosibirsk, Russia). A resulting product was used as a template in high-temperature 33 cycles of PCR. The length of a DNA probe was checked by electrophoresis in a 2% agarose gel.

### Labeling of DNA probes

DOP-PCR was used for fluorescent labeling of full chromosome DNA probes in the presence of MW-6 degenerate primer according to the previously published protocol (Artemov et al., 2015). We used 5-Tetramethylrhodamine-dUTP (Biosan, Novosibirsk, Russia) as a labeled nucleotide. The resulting probe was reprecipitated in 96% ethanol, and a DNA pellet was dissolved in 10–15 μl of a hybridization mixture (50% formamide, 10% sodium dextran sulfate, 2×SSC, 1% Tween 20).

### Fluorescence In Situ Hybridisation (FISH)

We checked the specificity of the X chromosome painting probe by FISH with air-dried chromosome preparations of *An*. *atroparvus*. Air-dried preparations of chromosomes were washed in 2×SSC at 37°C for 5 min three times. Then, they were dehydrated in 70%, 80%, and 96% ethanol for 5 min each at room temperature. After that, chromosomes were treated with a 100 μg/μl pepsin solution at 37°C, pH<7 for 10 min. Preparations were washed in 1×PBS twice for 5 min at room temperature. Then they were dehydrated again by the series of ethanol solutions (50%, 70%, 96%) for 5 min at room temperature and dried. The labeled DNA probe was dissolved in a hybridization mixture and placed on the chromosomal preparation, covered by a coverslip, and sealed with a universal adhesive “Moment-1” (Henkel, Moscow, Russia). Denaturation and hybridization steps were conducted in a programmable thermostat Thermobrite S500 (Beckman Coulter, Moscow, Russia) initially at 75°C for 15 min (denaturation) and at 37°C for 18 hours (hybridization). After hybridization, preparations were washed in a 50% formamide solution in 2×SSC at 45°C three times for 5 min followed by incubation in 2×SSC at 45°C for 5 min, 0.2×SSC at 45°C twice for 5 min, and 0.1×SSC at 45°C for 5 min. We applied DAPI (4’,6-diamidino-2-phenylindole) with antifade (Prolong Gold Antifade, ThermoFisher Scientific, Dia-m Company, Novosibirsk, Russia) on the surface of a dry preparation and visualized chromosomes with an AxioVision Z1 microscope (Carl Zeiss, OPTEC Company, Siberian Office, Novosibirsk, Russia).

### 3D-FISH

Females of *An*. *atroparvus* 23 hours post blood feeding were used for the experiment. Ovaries for 3D-FISH were extracted from 3 individual mosquitoes immediately before conducting hybridization. Ovaries were dissected in 1×PBS and were processed in accordance with the 3D-FISH protocol described in our previous study [19].

### 3D-immunostaining of nucleolus

We determined the location of nucleoli by immunostaining interphase nuclei with a fluorescently labeled antibody against fibrillarin, the basic component of the nucleolus fibrillar domain [20]. The material was extracted in the EBR solution (0.13M NaCl, 0.04M KCl, 0.018M CaCl_2_, 9mM HEPES) at +4°C and fixed in 4% of paraformaldehyde for 20 min at room temperature. Fixative solution was washed away by 1×PBS at room temperature three times for 5 min, and tissues were treated with the PBSTr solution (0.3% Triton-X100 in 1×PBS) for 30 min at room temperature. Then the material was incubated in block buffer (BB) (4% powdered milk, 10% FBS) for 30 min and then shaken in 1% solution of primary antibodies (Anti-fibrillarin [38F3], Abcam, Cambridge, UK) in BB at +4°C during the night. After that, tissues were washed in PBSTr three times for 15 min at room temperature. Staining was conducted in 0.25% solution of secondary antibodies Anti-Mouse IgG—FITC (Sigma-Aldrich, Dia-m, Novosibirsk, Russia) in BB at +4°C overnight. The washing step was performed in the same manner as above. Finally, the tissue was stained by DAPI (Prolong Gold Antifade, ThermoFisher Scientific, Dia-m Company, Novosibirsk, Russia) at +4°C for 8 hours.

### Image analysis

The series of the z-stack images obtained by a LSM 780 confocal microscope (Carl Zeiss, OPTEC Company, Siberian Office, Novosibirsk, Russia) and ZEN 2012 software (Carl Zeiss, OPTEC Company, Siberian Office, Novosibirsk, Russia) was processed and analyzed by three different softwares: (1) Fiji (ImageJ), (2) the complex of tools and plugins for the analysis of the nuclear spatial organization, TANGO [21], (3) and MongoDB database (MongoDB, Inc.). We used the “Mean” filter from the “Fast Filters 3D” plugin and the “Gaussian Blur 3D” filter from the “Misc Filters 3D” plugin for the pre-filter analysis step. We used the classical “thresholding” algorithm from the “Hysteresis Segmenter” plugin for the segmentation of NE (NE was expected as an imaginary surface, which covers the external voxels of the DAPI-labeled chromatin), X chromosome, and nucleolus. We conducted spatial measurements for each nucleus which was used in the statistical analysis by the “Simple Measure Geometrical” plugin. We employed the following parameters: Volume (in unit), Surface (in unit), Compacity, Feret, Elongation, DC measures from the “Simple Geometrical Measurements” plugin, and the minimum and maximum radial position parameters from the “Eroded volume fraction” plugin. The description of each parameter is available in the official manual of TANGO [22]. We also used other derived parameters in the statistical analysis such as:

- relative volume of the X chromosome CT:
  $$\%V_{rel}^{3}=V_{X}^{3}/V_{Nuc}^{3}\times 100,$$
  where $V_{X}^{3}$ is the volume of the X chromosome (μm^3^), $V_{Nuc}^{3}$ is the volume of the nucleus (μm^3^)
- α is the angle between longitudinal axis of X chromosome and tangent to the NE drown throw intersection point of longitudinal axis of X chromosome with NE:
  $$\alpha =asin(\frac{({EFV}_{max}–{EFV}_{min})\times {DC}_{nuc}}{{LD}_{x}}),$$
  where *EFV*_*max*_ and *EFV*_*min*_ are maximum and minimum radial positions of the X chromosome (%), *DC*_*nuc*_ and *LD*_*x*_ are the length of mean nucleus radius and longest axis of the X chromosome, respectively.

These parameters were applied for the measurement of nucleolus spatial organization. The complete data are provided in S1 Table and S2 Table. A statistical analysis was conducted with R programing language (The R Foundation) and RStudio IDE (RStudio, Inc.). We used a non-parametric Mann-Whitney U-test for sample comparisons. The results were considered significant when p<0.05. We employed a standard error in illustrations as a confidence interval.

## Results

### Visualization of the X chromosome and nucleolus in nuclei of *An*. *atroparvus*

**Fig 1:**
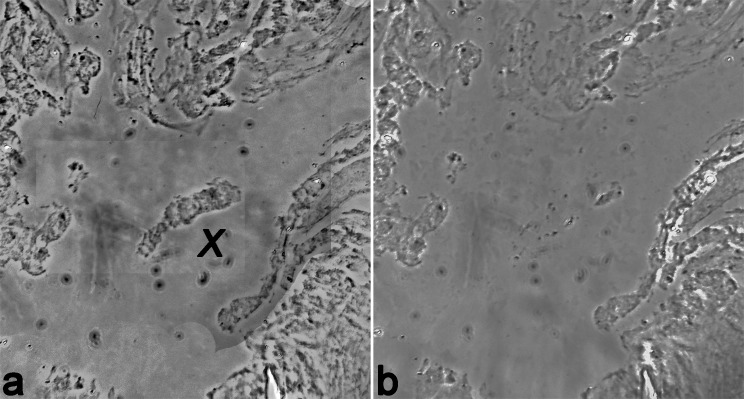
Microdissection of the *An*. *atroparvus* polytene X chromosome. Chromosome preparation from ovarian nurse cells before (a) and after (b) microdissection of the X chromosome.

CTs of X chromosomes in NC and FE cells were identified by *in situ* hybridization of the microdissected full X chromosome probe. We conducted microdissection of three individual X chromosomes from one ovary of *An*. *atroparvus*. Success of the procedure was confirmed by visual inspection of the preparation after microdissection (Fig 1).

**Fig 2:**
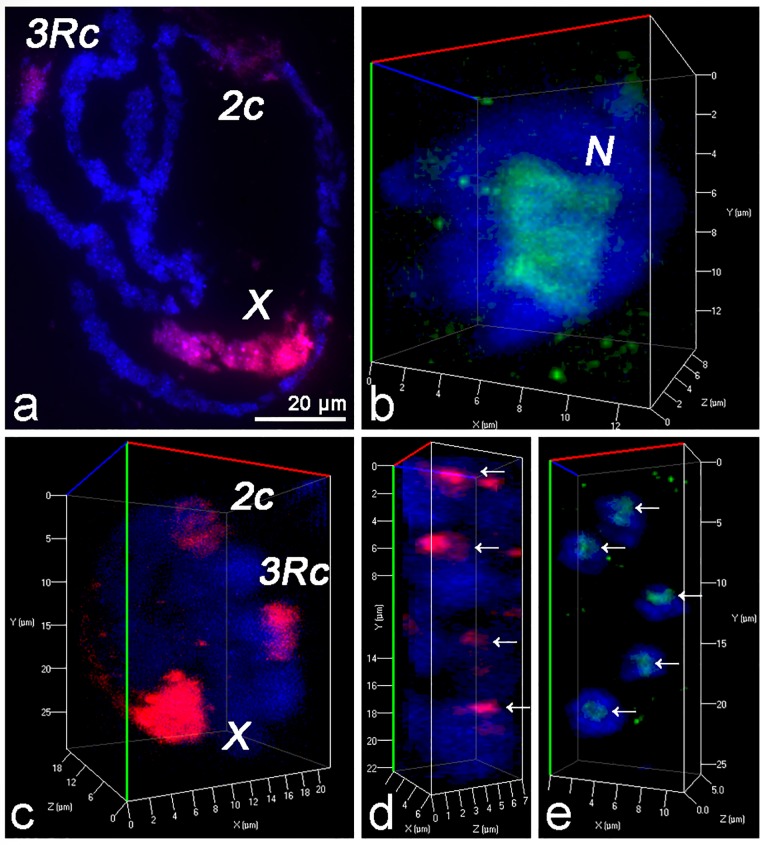
Visualization of the X chromosome and nucleolus in *An*. *atroparvus*. a) 2D-FISH of fluorescent X chromosome painting probe with squashed chromosome preparation from NCs. Specific labeling of the X chromosome (X) and unspecific labeling of pericentromeric regions of 3R (3Rc) and 2 (2c) are shown in red. b) Immunostaining of the nucleolus in an NC nucleus with a fluorescently labeled antibody against fibrillarin. The nucleolus (N) is shown in green. c) 3D-FISH of fluorescent X chromosome painting probe with an NC nucleus. d) 3D-FISH of fluorescent X chromosome painting probe with FE (arrows mark specific labeling of the X chromosome). e) Immunostaining of the FE nucleolus with a fluorescently labeled antibody against fibrillarin (arrows show specific labeling of the nucleolus). Chromosomes are stained with DAPI (blue).

FISH with chromosome preparations of NC confirmed specificity of obtained microdissection probes (Fig 2A). Pericentromeric heterochromatin regions of chromosomes 2 and 3 appeared non-specifically labeled by this probe. This non-specific hybridization can be explained by the presence of homologous repetitive DNA sequences in heterochromatin of the sex chromosome and autosomes. We visualized the nucleolus with a fluorescently labeled antibody against fibrillarin (Fig 2B). 3D FISH with chromosome preparations of NC also confirmed specificity of the X chromosome painting probe (Fig 2C). The volume and intensity of the signals from non-specifically labeled autosomal regions were much smaller than the same parameters for the X chromosome. 3D FISH with chromosome preparations of FE identified a single CT corresponding to the X chromosome (Fig 2D). Thus, the resulting microdissected painting probe allows adequate visualization of X chromosome CTs even in nonpolytenized interphase nuclei. We also successfully visualized the nucleolus with a fluorescently labeled antibody against fibrillarin in interphase nuclei of FE (Fig 2E).

### Tissue-specificity of the X chromosome relative volume in nuclei of An. atroparvus

**Fig 3:**
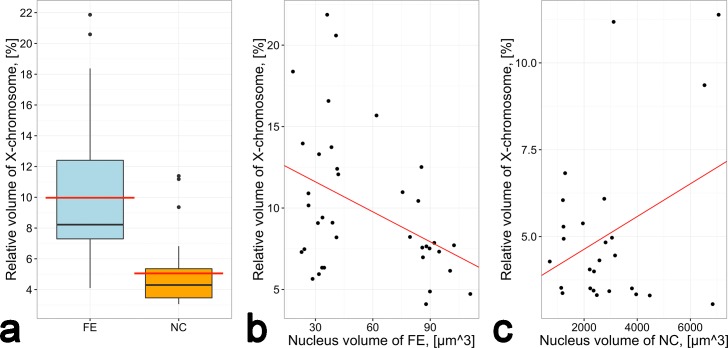
The relationship between the X chromosome volume and the nuclear volume in FE and NC. a) The volume of the X CT relative to the nuclear volume in FE and NC. The red line is a mean. b) Weak significant negative correlation between the volume of the X CT and the nuclear volume in FE. The red line marks a fitted linear model, r = −0.41. c) Weak non-significant positive correlation between the volume of the X CT and the nuclear volume in NC. The red line marks a fitted linear model, r = 0.35.

The volume of the X chromosome relative to the nuclear volume is significantly greater in FE (9.97%) than in NC (5.05%) (p = 1.733e-08, Mann-Whitney U-test) (Fig 3A). There is a weak significant negative correlation between the X chromosome volume and the nuclear volume in FE (r = −0.41, p<0.05, Pearson test) (Fig 3B), but there is a weak non-significant positive correlation between the X chromosome volume and the nuclear volume in NC (r = 0.35, p>0.05, Pearson test) (Fig 3C). Thus, unlike NC, the X CT does not contribute to the growth of the FE nucleus. In contrast, the volume of the nucleolus is in direct proportion to the nuclear volume in both tissues.

The shape of the X CT, which is expressed in terms of a standard deviation (DC) of the mean radius, elongation, and roundness, varies identically in both cell types. We found no statistically significant differences between the cell types.

### Peripheral location of the X chromosome and nucleolus

**Fig 4:**
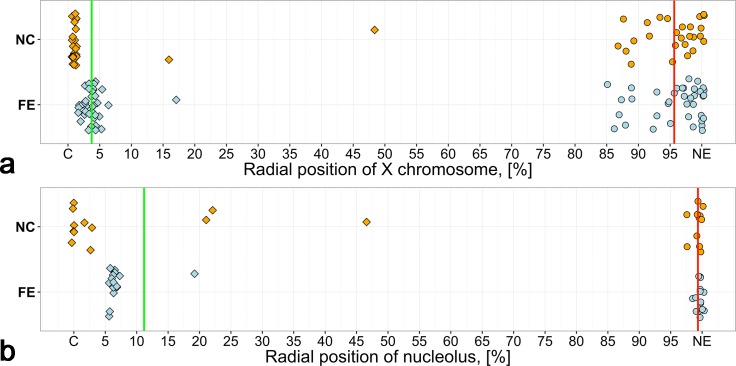
Spatial positions of the X chromosome and nucleolus with respect to the nuclear periphery. a) Radial positions (%) of X chromosomes in NC and FE. b) Radial positions (%) of nucleoli in NC and FE. Rhombi correspond to individual values of minimum radial positions, circles correspond to individual values of maximum radial positions. Red lines mark the mean of maximum radial positions, and green lines mark the mean of minimum radial positions. C—the center of the nuclei, NE–nuclear envelope.

The mean of maximum values of the X chromosome radial position is 95.65% meaning that the contact between the X chromosome and the NE is permanent in NC and FE (Fig 4A) in accordance with the previous study [16]. Nucleolus has also frequent contacts with the NE in both tissues (Fig 4B). These contacts are independent of the nucleolus shape and volume, which vary widely. The maximum values of the radial position of the X chromosome and nucleolus are not significantly different between NC and FE (p = 0.6567 for the X chromosome and p = 0.3229 for the nucleolus, Mann-Whitney U-test). However, the minimum values of the radial position of the X chromosome and nucleolus are significantly smaller in NC than in FE (p = 3.238e-09 for the X chromosome, and p = 0.02903 for the nucleolus, Mann-Whitney U-test) meaning that both the X chromosome and nucleolus are located closer to the center of the nucleus in NC compared with FE. This observation could provide indirect support for the greater involvement of X chromosome and nucleolus in transcription in NC than in FE.

### Tissue-specific dynamics of the X chromosome location during nuclear growth

**Fig 5:**
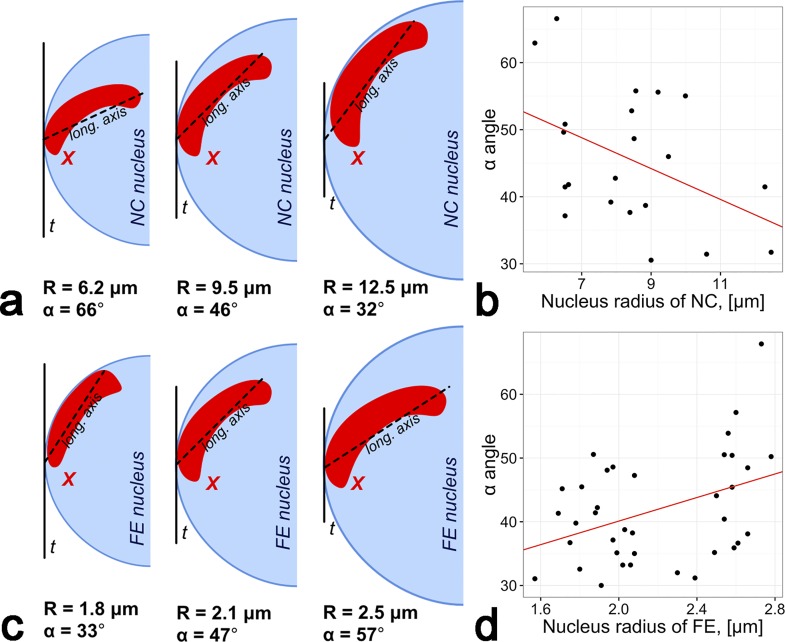
Tissue-specific dynamics of the X chromosome location during nuclear growth. a) A scheme demonstrating the decrease of the longitudinal axis of the X chromosome related to the nucleus growth in NC. b) Weak (r = -0.43) significant (p = 0.001) negative correlation between nucleus radius and α angle (°) in NC. c) A scheme demonstrating the increase of the longitudinal axis of the X chromosome related to the nucleus growth in FE. d) Weak (r = 0.39) significant (p = 0.01) positive correlation between nucleus radius and α angle (°) in FE. t—tangent to NE, R—the nucleus radius.

Quantitative characteristics of spatial relationships between the X chromosome and the NE were studied with the help of an α angle (see Materials and Methods). The mean value of α in both tissues corresponds to the position of the X chromosome when its longitudinal axis is parallel to the NE. However, some nuclei were characterized by α = 67°. In this case, the longitudinal axis was directed toward the intranuclear space. We found the relationship between this parameter and the nuclear volume. The increase of the mean nuclear radius correlated with the decrease of the α angle in NC (Fig 5A and 5B). Thus, during the nucleus growth in NC, the X CT moves toward the NE. There was an inverse trend in FE (Fig 5C and 5D). One of the chromosome ends moved from the NE to the intranuclear space during the nucleus growth in FE.

## Discussion

### Tissue-specificity of the X chromosome spatial organization

Previous work showed that FE and NC differ by X and 3R chromosome interposition in *An*. *messeae* [16], which could be explained by the chromocenter formation in somatic cells [14]. Here, we were not able to detect significant differences in the shape of the X chromosome or nucleolus between nuclei of NC and FE. However, we identified tissue-specific features of the X chromosome relative size, suggesting different chromatin organization in the X chromosome and/or expression level of the X chromosome genes in NC compared with FE. Previously described tissue-specific differences in the X chromosome attachment to the NE [14,16] have been further explored in this work using the new computational tool TANGO [21]. Relative size parameter depends on the correctness of the segmentation algorithm, the accuracy of which is deteriorated due to varying signal-to-noise ratios in photomicrographic images. To overcome this problem, we visually monitored the quality of the segmentation for each nucleus. In some cases, we used the “hessian transform” pre-filter for the segmentation quality improvement in accordance with the recommendations of TANGO developers. The tissue-specific differences of the dynamics of the X chromosome could be associated with the difference in the level of polyteny. Indeed, chromosome in NC are polytene, and chromosome in FE are non-polytene. Nevertheless, tissue-spcecific features in the 3D genome organization could result in gene expression differences [1, 10, 13].

### Dynamics of the nuclear spatial organization

Based on the above described data (the displacement of the longitude axis of the X chromosome during the nucleus growth, the peripheral location of the X chromosome), we can assume that “activation” of NE-attachment regions of the X chromosome in NC and FE occurs gradually depending on the development stage. Various phases of NC and FE development can be characterized by different numbers of X chromosome attachment regions. In the late developmental stages, the pericentric region of the X chromosome in NC has a strong NE-attachment region [14,16]. There are at least 3 major lamin-binding regions along the chromosome, which potentially form NE-attachments (data not shown). The pericentric region is permemantly attached to the NE, but the other attachments are being “turned on” during development, moving telomeric end from the nuclear interior to periphery. On the contrary in FE, the same attachments are being “turned off” during development, moving telomeric end from the periphery to the nucleus center. These movements could be connected with transcription activation/inactivation of distinct chromosome segments. Some chromosome movements can be forced by the change of the size of nucleolus. However the gradual increase of nucleoli size in both NC and FE cannot be the reason for different X chromosome movement in these cell types.

Dynamism of polytene chromosome attachments to the NE has been shown by the modeling of the 3D organization of the salivary glands interphase nucleus of *Drosophila*. Fourteen of 15 known high-frequency contacts of chromosomes with NE have been described as intercalary heterochromatin, and one is a region of late replication [23]. A computational analysis has found 33 additional sub-high-frequency chromosome attachments with the NE [24]. Twenty new attachment regions corresponded to intercalary heterochromatin, and 5 were regions of late replication. However, 3 of these attachments corresponded to euchromatin [24]. These results suggest that affinity for the NE can change gradually, with the highest affinity for the NE almost exclusively possessed by intercalary heterochromatin, and the next highest affinity for the NE mostly a property of intercalary heterochromatin. What is the effect of chromosome-NE attachments on the nucleus architecture? Computer modeling demonstrates that a nucleus with the most numerous attachments of chromosomes to the NE form more precise chromosome territories with fewer intersections between chromosomes [25]. Intra-arms contacts happen more often in a nucleus with more NE-attachment regions in comparison with a nucleus which does not contain specific attachments. At the same time, the contacts between different arms happen more rarely in nuclei with more numerous NE-attachments [25]. If chromosome-NE attachments are gradually “turned on” with an increase in the volume of the NC nucleus, we can expect a decrease in the frequency of contacts involving the X chromosome with other chromsomes.

## Conclusion

Principles of the 3D genome organization must be thoroughly studied in vector species because of possible dynamic changes in the nuclear architecture upon infection with a pathogen [17]. Previously we demostrated tissue-specific features of the spatial chromosome organization in *An*. *messeae* based on data obtained by manual geometrical measurements of only two points for every nucleus [16]. In this work we have shown that the methods of the chromosome spatial organization analysis using TANGO is applicable to studying the shape and size of polytene chromosomes and chromosome dynamics during nucleus growth. The movement of the longitudinal axis of the X chromosome with the change of the nucleus size is likely associated with the change in the number of NE-attachment regions. This idea agrees well with the data obtained by modeling of *Drosophila* salivary gland nuclei [23–24]. The tissue-specific differences of the dynamics of the X chromosome and NE-attachment regions could result in gene expression differences [1, 10, 13]. Spatial characteristics of the X chromosome and nucleolus in *An*. *atroparvus* will serve as a baseline for similar studies in other species from the *Maculipennis* complex to which *An*. *atroparvus* belongs. Future studies will also address species-specific aspects of the nuclear architecture. A study of the evolution of the nuclear architecture will assess the possibility of using some features of 3D genome organization as markers for understanding phylogenetic relationships within the species complex.

## Supporting information

S1 Table Source data with observations and corresponding parameters of nucleus and X chromosome exporting from TANGO. (CSV)

S2 Table Source data with observations and corresponding parameters of nucleus and nucleolus exporting from TANGO. (CSV)

## Abbreviations

2D: two-dimensional
3D: three-dimensiona
BB: block buffer
CT(s): chromosome territory(ies)
DAPI: 4',6-diamidino-2-phenylindole
DC: standard deviation
DOP-PCR: degenerate oligo primer polymerase chain reaction
EBR: Ephrussi-beadle ringer
FBS: fetal bovine serum
FISH: fluorescence *in situ* hybridisation
FE: follicular epithelium cells
LAD(s): lamin-associated domain(s)
N: nucleolus
NAD(s): nucleolus associated domain(s)
NC(s): nurse cells
NE: nuclear envelope
NOR(s): nucleolus organizer region(s)
PBS: phosphate buffered saline
PBSTr: phosphate buffered saline with 0,3% Triton-X100
SSC: saline sodium citrate

## References

[1] Fritz AJ, Barutcu AR, Martin-Buley L, Van Wijnen AJ, Zaidi SK, Imbalzano AN, et al Chromosomes at Work: Organization of Chromosome Territories in the Interphase Nucleus. J Cell Biochem. 2016;117(1):9–19. 10.1002/jcb.25280 26192137 PMC4715719

[2] Cremer T, Cremer M. Chromosome territories. Vol. 2, Cold Spring Harbor perspectives in biology. 2010. 10.1101/cshperspect.a003889 PMC2829961 20300217

[3] Paz N, Felipe-Blanco I, Royo F, Zabala A, Guerra-Merino I, García-Orad Á, et al Expression of the DYRK1A gene correlates with its 3D positioning in the interphase nucleus of Down syndrome cells. Chromosom Res. 2015;23(2):285–98. 10.1007/s10577-015-9467-7 25645734

[4] Goldman RD, Gruenbaum Y, Moir RD, Shumaker DK, Spann TP. Nuclear lamins: Building blocks of nuclear architecture. Vol. 16, Genes and Development. 2002 p. 533–47. 10.1101/gad.960502 11877373

[5] Prieto J-L, McStay B. Nucleolar biogenesis: the first small steps. Biochem Soc Trans. 2005;33(Pt 6):1441–3. 10.1042/BST20051441 16246141

[6] Gatti M, Pimpinelli S. Functional elements in *Drosophila melanogaster* heterochromatin. Annu Rev Genet. 1992;26(1):239–76. 1482113 10.1146/annurev.ge.26.120192.001323

[7] Marchi A, Pili E. Ribosomal RNA genes in mosquitoes: localization by fluorescence in situ hybridization (FISH). Heredity (Edinb). 1994;72 (Pt 6):599–605. 7914517 10.1038/hdy.1994.83

[8] Sullivan GJ, Bridger JM, Cuthbert AP, Newbold RF, Bickmore WA, McStay B. Human acrocentric chromosomes with transcriptionally silent nucleolar organizer regions associate with nucleoli. EMBO J. 2001;20(11):2867–77. 10.1093/emboj/20.11.2867 11387219 PMC125486

[9] Cremer M, Küpper K, Wagler B, Wizelman L, Hase JV, Weiland Y, et al Inheritance of gene density-related higher order chromatin arrangements in normal and tumor cell nuclei. J Cell Biol. 2003;162(5):809–20. 10.1083/jcb.200304096 12952935 PMC2172812

[10] Parada LA, McQueen PG, Misteli T. Tissue-specific spatial organization of genomes. Genome Biol. 2004;5(7):R44 10.1186/gb-2004-5-7-r44 15239829 PMC463291

[11] Meuleman W, Peric-Hupkes D, Kind J, Beaudry JB, Pagie L, Kellis M, et al Constitutive nuclear lamina-genome interactions are highly conserved and associated with A/T-rich sequence. Genome Res. 2013;23(2):270–80. 10.1101/gr.141028.112 23124521 PMC3561868

[12] Hochstrasser M, Sedat JW. Three-dimensional organization of *Drosophila melanogaster* interphase nuclei. I. Tissue-specific aspects of polytene nuclear architecture. J Cell Biol. 1987;104(6):1455–70. 3108264 10.1083/jcb.104.6.1455 PMC2114489

[13] Hochstrasser M, Sedat JW. Three-dimensional organization of *Drosophila melanogaster* interphase nuclei. II. Chromosome spatial organization and gene regulation. J Cell Biol. 1987;104(6):1471–83. 3108265 10.1083/jcb.104.6.1471 PMC2114521

[14] Stegniĭ VN. Systemic reorganization of the architectonics of polytene chromosomes in the onto- and phylogenesis of malaria mosquitoes. Genetika. 1987;23(5):821–7. 3623084

[15] Stegniĭ VN, Sharakhova MV. Systemic reorganization of the architectonics of polytene chromosomes in onto- and phylogenesis of malaria mosquitoes. Structural features regional of chromosomal adhesion to the nuclear membrane. Genetika. 1991;27 (5):828–35. 1916252

[16] Artemov G, Bondarenko S, Sapunov G, Stegniy V. Tissue-specific differences in the spatial interposition of X-chromosome and 3R chromosome regions in the malaria mosquito *Anopheles messeae* Fall. PLoS One. 2015;10(2). 10.1371/journal.pone.0115281 PMC4324937 25671311

[17] Sharakhov IV, Sharakhova MV. Heterochromatin, histone modifications, and nuclear architecture in disease vectors. Curr Opin Insect Sci. 2015;10:110–7. 10.1016/j.cois.2015.05.003 26097808 PMC4470418

[18] Artemov GN, Stegnii VN. Molecular genetic analysis of the X-chromosomal nuclear envelope attachment region in nurse cells of the malaria mosquitoes *Anopheles messeae* Fall. Genetika. 2011;47(10):1307–14. 22232918

[19] Kokhanenko AA, Anan’ina TV, Stegniy VN. The changes in chromosome 6 spatial organization during chromatin polytenization in the *Calliphora erythrocephala* Mg. (Diptera: Calliphoridae) nurse cells. Protoplasma. 2013;250(1):141–9. 10.1007/s00709-012-0385-7 22322965

[20] Ochs RL, Lischwe MA, Spohn WH, Busch H. Fibrillarin: a new protein of the nucleolus identified by autoimmune sera. Biol Cell. 1985;54(2):123–33. 2933102 10.1111/j.1768-322x.1985.tb00387.x

[21] Ollion J, Cochennec J, Loll F, Escudé C, Boudier T. TANGO: A generic tool for high-throughput 3D image analysis for studying nuclear organization. Bioinformatics. 2013;29(14):1840–1. 10.1093/bioinformatics/btt276 23681123 PMC3702251

[22] TANGO. Tango—Tools for Analysis of Nuclear Genome Organisation | MeasureGeometrical. Available: http://biophysique.mnhn.fr/tango/measuregeometrical

[23] Hochstrasser M, Mathog D, Gruenbaum Y, Saumweber H, Sedat JW. Spatial organization of chromosomes in the salivary gland nuclei of *Drosophila melanogaster*. J Cell Biol. 1986;102(1):112–23. 3079766 10.1083/jcb.102.1.112 PMC2114037

[24] Kinney NA, Sharakhov IV, Onufriev AV. Investigation of the chromosome regions with significant affinity for the nuclear envelope in fruit fly–A model based approach. PLoS One. 2014;9(3). 10.1371/journal.pone.0091943 PMC3961273 24651400

[25] Kinney NA, Onufriev AV, Sharakhov IV. Quantified effects of chromosome-nuclear envelope attachments on 3D organization of chromosomes. Nucleus. 2015;6(3):212–24. 10.1080/19491034.2015.1056441 26068134 PMC4615791

